# The bacterial burden on computer keyboards across selected university facilities at Liverpool John Moores University, City Campus, Byrom Street, Liverpool

**DOI:** 10.1371/journal.pone.0324977

**Published:** 2025-06-11

**Authors:** Emmanuel Oladipo Babafemi, Hannah Church

**Affiliations:** 1 School of Pharmacy and Biomolecular Sciences, Liverpool John Moores University, Liverpool, United Kingdom; 2 Biomedical Science, School of Pharmacy and Biomolecular Sciences, Liverpool John Moores University, Liverpool, United Kingdom; Universidade dos Açores Departamento de Biologia: Universidade dos Acores Departamento de Biologia, PORTUGAL

## Abstract

**Background:**

Bacterial burden within universities is important to investigate due to the high footfall of both students and staff, who may bring contaminants from body fluids, food, and the environment. These bacterial contaminants may be harmful to both immunocompromised and immunocompetent individuals. A focus on online resources and communication with workplaces and universities bring an important issue of computer keyboards and mice becoming potential reservoirs for such bacteria.This study aimed to investigate the presence of bacteria on computer keyboards and mice across the Liverpool John Moores University campus by detecting the presence of bacterial growth, identifying these bacteria and the role of disinfection in lowering the bacterial counts and changing their diversity.

**Materials and method:**

A total of 478 pre- and post-treatment swab samples were taken using sterile cotton swabs moistened in sterile nutrient broth (Thermo Scientific™ Oxoid™) from both the keyboards and mice of facilities across the university campus were cultured at 37°C on blood agar plates. A total period of 30 minutes elapsed between first sampling (pre-treatment) and disinfection before the second sampling (post-treatment). Identification of isolated bacterial species was done with Matrix-Assisted Laser Desorption/Ionization Time-of-Flight (MALDI-TOF) mass spectrometry according to protocol by BioMérieux VITEK® MS prime.

**Results:**

One hundred and fifty-four (64.4%) of pre-treatment and Forty-nine (20.5%) of post-treatment cultures showed microbial growth, which were identified as various species of Gram-positive cocci, Gram positive bacilli and Gram-negative cocci.

**Discussion:**

Across the Liverpool John Moores campus computer keyboards and mice were found to be contaminated with many bacteria, all of which were identified as opportunistic and therefore are not likely to cause harm to healthy individuals. However, there is a greater risk of infection to immunocompromised individuals, which could lead to co-morbidities.

## Introduction

The bacterial burden on surfaces has been researched for many years in hospitals with an effort to reduce bacterial contamination and infections leading to co-morbidities [1,2,3]. Assessment of the presence of bacteria should be applied to universities and workplaces to protect students and staff from harmful pathogens they may be unknowingly spreading [4,5,6,7]. Bacteria can be spread via direct and indirect contact with infected droplets, airborne particles and fomites, with many bacterial species able to survive on inanimate surfaces for weeks to even months [8,9].

Due to increased online presence within universities such as the utilization of online resources for announcements, accessing learning content, contacting staff/students and handing in/marking work, computers have become one of the most used interfaces for both students and staff. Computer keyboards and mice have the most contact with a person’s hands, with average keyboards having over 100 keys and many grooves. This increases the surface area and creates difficult to reach recesses which results in keyboards being problematic and time consuming to thoroughly clean, therefore they may harbour bacterial pathogens [4]. It is important to consider that bacteria present may be opportunistic pathogens meaning they are not harmful to immunocompetent individuals, however, may pose a risk of infection to immunosuppressed individuals.

This study will assess the bacterial burden of multi-user computer keyboards and mice across the Liverpool John Moores city campus (LJMU), identify the bacterial species grown from the samples and discuss whether disinfection using a 70% isopropyl alcohol wipe (PDI Sani-Cloth^®^) is sufficient to reduce the presence of bacteria within the university campus.

## Methods

Computer keyboards and mice were sampled from across the Liverpool John Moores University’s City campus with a total of 478 swabs taken between 9^th^ −20^th^ January 2023–8^th^ −19^th^ January 2024 across different facilities on LJMU campus (see Table 1). Initially computer keyboard and mouse samples were taken with a sterile cotton swab (SLS® Select Swab Woodstick with Cotton Tip Single in Peel Pouch), which was then capped and given an identification number denoting the sample was part of the pre-treatment group. During sampling, the mouse was swabbed first because this is the part of the computer that has the most contact with our hands. Then the keyboard was sampled with the same cotton swab by rubbing it along the top of the key. Lastly, the swab was dragged along the grooves of the keyboard to ensure the whole keyboard was thoroughly sampled. Immediately, a second sample was taken from the computer using a new swab after the keyboard and mouse had been disinfected with a 70% v/v isopropyl alcohol wipe (PDI Sani-Cloth®). A total period of 30 minutes elapsed between first sampling and disinfection before the second sampling (post-treatment) This sample was then capped and given identification number denoting it was a post-treatment sample. 239 computer samples were taken (see Table 1), with computers in various environments around campus including the library (69 computers), lecture theatres (14 computers), IT offices (80 computers), IT Suites (70 computers), communal areas (6 computers). The swabs were transferred to a laboratory where they were inoculated onto blood agar plates (Thermo Scientific™) and incubated aerobically overnight at 37°C [10, 11, 12] using Thermo Scientific™ incubator. For the initial stages of the investigation colony growth, gram staining [13,14,15] and morphology assessment was performed to group the bacterial species found. A selection of these samples was sub cultured and incubated overnight using Thermo Scientific™ incubator prior to analysis using MALDI-TOF spectrometry according to protocol by BioMérieux VITEK® MS prime to identify bacterial species [16,17,18] using α-Cyano-4-hydroxycinnamic acid (CHCA) as a matrix [19]. The BioMérieux VITEK® MS prime algorithm enables accurate discrimination of all species identified with performance ranging from 98 to 100%.

**Table 1:** Resut showing the colonial growth from pre- and post-treatment cultures.

Keyboard/mice	Total (n = 239)		Total (n = 239)		Pre-treatment outcomes			Post-treatment outcome		
**Locations (City campus)**	**Pre-treatment**		**Post-treatment**	
	**Growth**	**No Growth**	**Growth**	**No Growth**	**GPC**	**GPB**	**GNC**	**GPC**	**GPB**	**GNB**
Library	53	16	4	65	52	6	0	4	0	0
Lecture theatres	9	5	2	12	5	4	0	0	2	0
IT offices	20	60	1	79	11	9	0	1	0	0
IT Suites	69	1	40	30	22	14	2	24	5	0
Communal areas	3	3	2	4	2	2	0	0	1	0
Total	154	85	49	190	92	35	2	2	8	0	

Key: n, frequency; GPC, Gram-positive cocci; GPB, Gram-positive bacilli; GNC, Gram-negative cocci.

## Results

**Fig 1:**
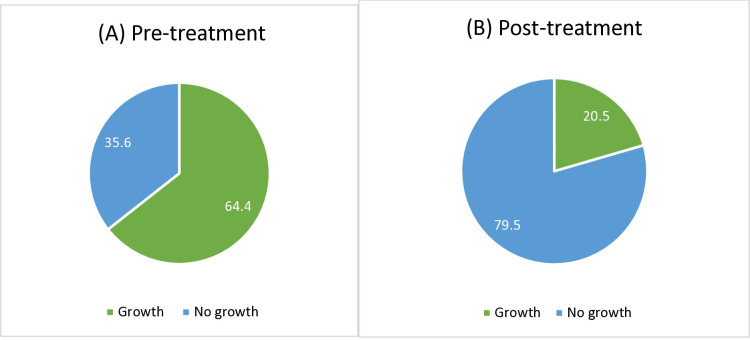
The percentage growth and no growth from cultures from pre and post treatment samples.

Growth on the blood agar plates demonstrated One hundred and fifty-four (64.4%) of pre-treatment samples (A) had microbial colony growth, decreasing to Forty-nine (20.5%) among the post-treatment samples (B) group as can be seen in Fig 1.

**Fig 2:**
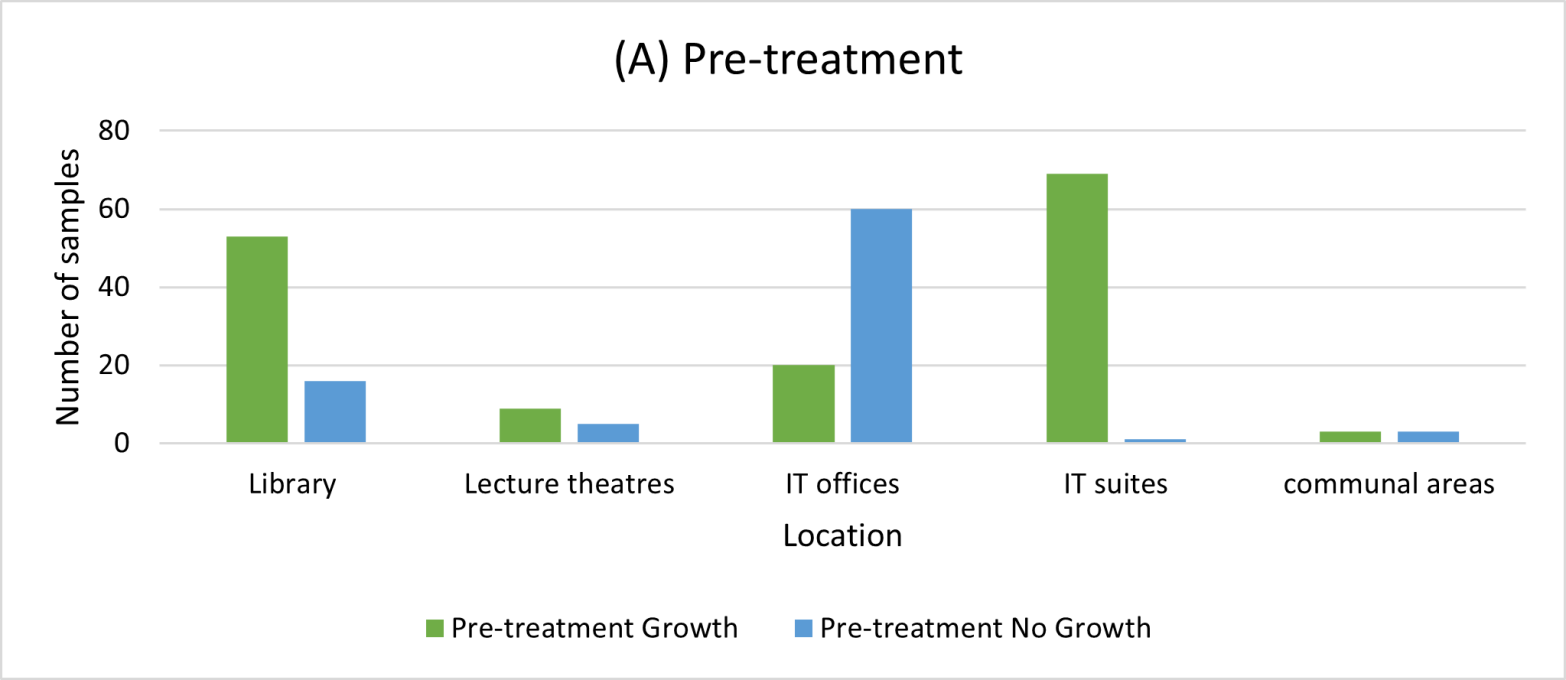
The bacterial growth distribution for pre-treatment samples across the locations.

The distribution of this growth indicated that library and IT suites samples produced the greatest number of samples with growth, 53 and 69 samples respectively as can be seen in Fig 2.

Further investigation, using Gram staining and MALDI-TOF MS (see Table 1) indicated a high proportion of the bacterial growth present was identified as Gram-positive cocci (GPC) species such as *Staphylococcus albus*, *S. epidermidis*, *S. hominis*, *S. saprophyticus*, *S. warneri* and *Micrococcus luteus*, with ninety-two (71%) of pre-treatment and twenty-nine (78%) of post-treatment growth resulting from GPC species (see Tables 1 and 2).

**Table 2:** Identification of bacterial isolates using MALDI-TOF BioMérieux VITEK® MS prime.

Keyboards/mice	Identification using Matrix-Assisted Laser Desorption/Ionization Time-of-Flight (MALDI-TOF BioMérieux VITEK® VITEK® MS prime)			
Locations (city campus)	Identification (Pre-treatment)		Identification (Post treatment)	
	Name of bacteria	Frequency (n)	Name of bacteria	Frequency (n)
Library	*Staphylococcus epidermidis*	14^*1^	*Staphylococcus epidermidis*	3^*1^
	*Staphylococcus albus*	8^*1^	*Staphylococcus albus*	1^*1^
	*Staphylococcus hominis*	12^*1^	–	–
	*Micrococcus luteus*	9^*1^	–	–
	*Bacillus subtilis var. amyloliquefaciens*	2^*2^	–	–
	*Bacillus licheniformis*	4^*2^	–	–
	*Staphylococcus saprophyticus*	4^*1^	–	–
Lecture theatres	*Staphylococcus warneri*	1^*1^	*Bacillus licheniformis*	2^*2^
	*Bacillus licheniformis*	3^*2^	–	–
	*Staphylococcus hominis*	3^*1^	–	–
	*Bacillus cereus*	1^*2^	–	–
	*Staphylococcus cohnii spp urealyticus*	1^*1^	–	–
IT offices	*Staphylococcus capitis*	4^*1^	*Bacillus cereus*	1^*2^
	*Staphylococcus warneri*	3^*1^	–	–
	*Staphylococcus epidermidis*	4^*1^	–	–
	*Bacillus cereus*	6^*2^	–	–
	*Bacillus pumilus*	3^*2^	–	–
IT Suites	*Staphylococcus hominis*	9^*1^		
	*Staphylococcus haemolyticus*	7^*1^	–	–
	*Moraxella osloensis*	2^*3^	*Moraxella osloensis*	2^*3^
	*Staphylococcus warneri*	6^*1^	–	–
	*Bacillus subtilis var. amyloliquefaciens*	5^*2^	*Bacillus subtilis var. amyloliquefaciens*	4^*2^
	*Bacillus licheniformis*	9^*2^	*Bacillus licheniformis*	1^*2^
Communal areas	*Bacillus licheniformis*	2^*2^	*Bacillus licheniformis*	2^*2^
	*Staphylococcus albus*	2^*1^	–	–
Total		124		16

**Key**: (n), frequency of bacteria identified;

*1 Gram positive cocci;

*2 Gram positive bacilli;

*3 Gram Negative coccobacilli

Gram-positive bacillus (GPB) bacteria were also isolated from samples from both pre- and post- treatment swabs, with thirty-five (27%) of pre-treatment and eight (23%) of post-treatment growth being from GPB species. Examples of GPB species found include *Bacillus licheniformis*, *B. subtilis var. amyloliquefaciens* and *B. cereus*, which are usually present in soil [10]. Lastly, two (1.6%) of pre-treatment growth was Gram-negative cocci (GNC) species identified as *Enhydrobacter aerosaccus* and *Moraxella osloensis* as shown in Table 2.

## Discussion

Hand hygiene is the primary action to prevent infection and reduce the spread of multi-resistant organisms [20]. Numerous studies have indicated that computer keyboards (and mice) can become contaminated with pathogenic bacteria. As with health care settings, computer keyboards in educational institutions may act a mechanism for the transmission of pathogenic bacteria. Previous studies have demonstrated that other shared communication equipment, such as telephones, can also become contaminated by potentially pathogenic microorganisms, often members of the human microbiota [21,22].

Overall, the results showed that there were bacteria present on the computer keyboards and mice with 64.4% of the pre-treatment sample having colonial growth. This result is consistent with studies from UK and India [23,24]. It was expected that this would be higher considering the high footfall of both students and staff through the area where the samples were taken. Some factors could account for samples not showing microbial growth due to inability to provide anaerobic conditions. The samples were subject to aerobic conditions, where only species supported in this environment would grow. Therefore, if a second culture had been incubated anaerobically or in microaerophilic conditions there may have been a higher percentage of viable cultures. Another factor to consider is lasting vigilance of hygiene standards from the COVID-19 pandemic, in addition to continued presence of hand sanitiser and cleaning products available around the university campus.

All the bacteria identified from samples are commonly found as part of the normal human flora of the skin, mucus membranes and respiratory tract. These species identified are all opportunistic pathogens, which do not usually cause disease in healthy individuals however the presence of these microorganisms poses a greater risk to individuals who are immunosuppressed or compromised [4]. The GPC species found are a part of normal human microbiota of the skin and nasal passage; however, these are also opportunistic pathogens that are associated with many diseases and co-morbidities especially in individuals that are immunosuppressed [25]. It was expected that these species would be present; however, it is surprising that pathogenic bacteria were not isolated due to the number and variety of individuals using the facilities.

**Fig 3:**
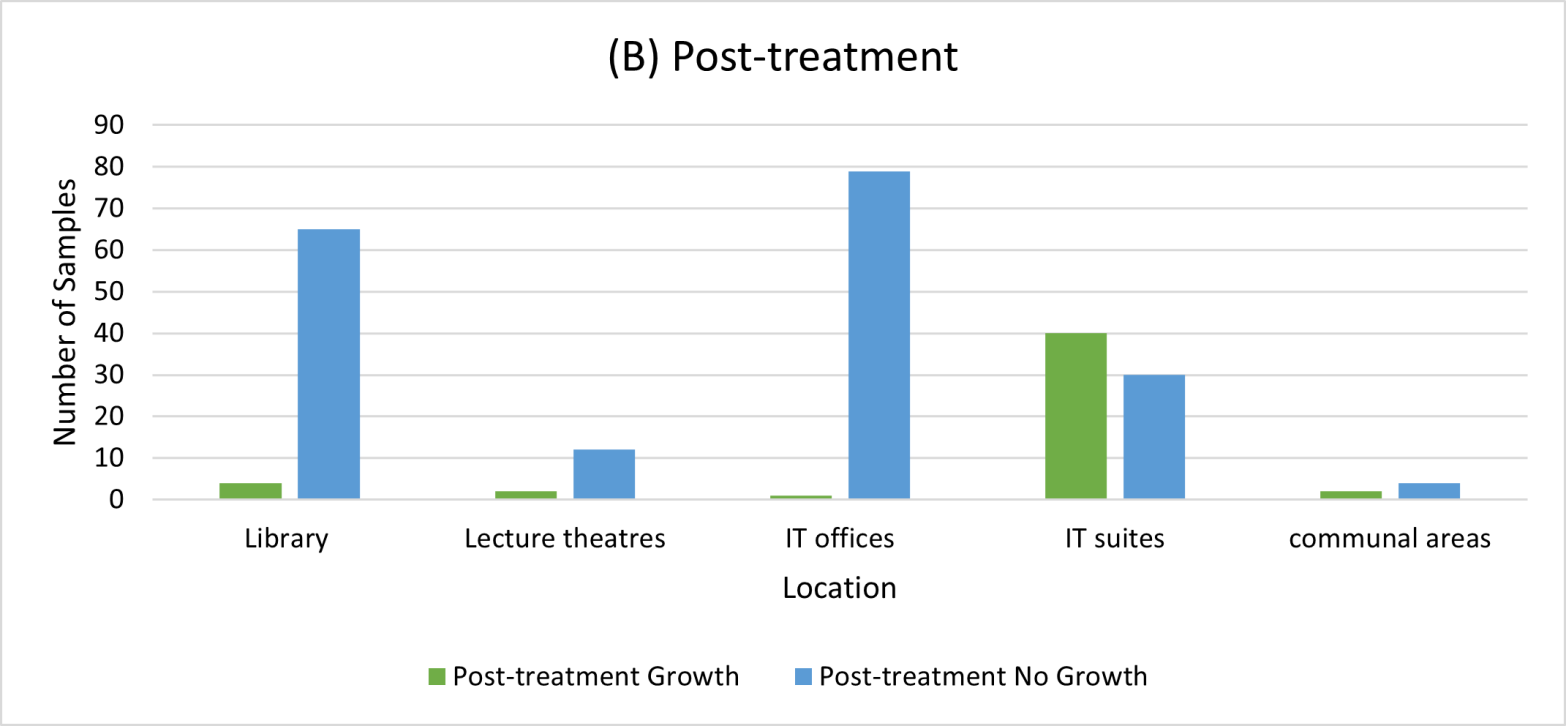
The bacterial growth distribution for post-treatment samples across the locations.

This percentage of no growth increased from 35.6% in the pre-treatment group to 79.5% in the post-treatment group as shown in (Fig 3), suggesting that the bacteria burden was decreased with the use of 70% isopropyl alcohol wipes confirming the effectiveness of the intervention.

Further research with a larger number of samples and a wider sampling area including a collaborative effort across different universities should be considered. Improvement to the method should be considered including the use of multiple different selective and differential agar plates to promote the growth of more varied bacteria [10,26]. Incubating a repeat sample in anaerobic conditions could help to assess the growth of different species of bacteria that may be present.

## Conclusion

This study shows that bacterial contamination of computer mice and computer keyboards is prevalent across different facilities in the university. The commonest bacteria are commensal skin organisms. The study also demonstrates that Gram positive bacteria are mostly isolated from the computer mice and computer keyboards, and a few Gram-negative bacteria. The study emphasises the importance of adequate decontamination procedures using swipe (disinfectant) with a drastic reduction in the number of bacteria isolated post-treatment. We strongly recommend from the outcomes of the study that methods of disinfecting the computer mice and computer keyboards should be undertaken by every user of the computer before and after the usage of the computer mice and the computer keyboards. These approaches if undertaken properly will reduce to the barest minimum the bacterial burden on the University computer mice and computer keyboards.

## Supporting information

S1 Table Minimal data set-MALDI-TOF Results. (TIF)
