# Supplementary figures and images for: The bacterial burden on computer keyboards across selected university facilities at Liverpool John Moores University, City Campus, Byrom Street, Liverpool
Source: PLoS One. 2025 Jun 11;20(6):e0324977. doi: 10.1371/journal.pone.0324977 (PMC12157171; doi:10.1371/journal.pone.0324977)

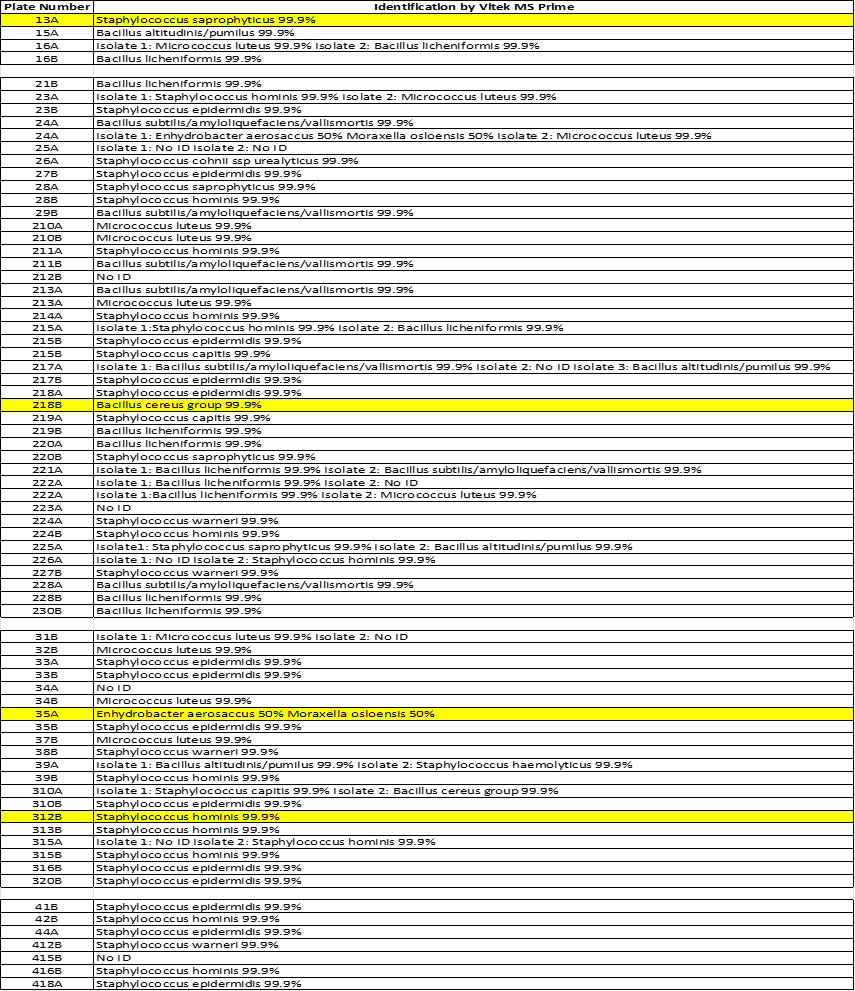

Supplement: S1 Table — (TIF) [file pone.0324977.s001.tif]
